# Supplementary material for: Effect of low-dose radiation pre-irradiation on postoperative local chest wall recurrence of breast cancer—A retrospective study
Source: PeerJ. 2025 Jan 2;13:e18717. doi: 10.7717/peerj.18717 (PMC11700491; doi:10.7717/peerj.18717)
Supplement: Supplemental Information 1 [file peerj-13-18717-s001.zip › data/Table 1-4/Table 1-4.docx]

**Table 1 The clinical characteristic of two group patients**

| **Characteristics** | **LDR Group（n）** | **No LDR Group（n）** | ***p*** |
| --- | --- | --- | --- |
| **Median age** (range) | 50 (24-69) | 50 (25-70) | 0.982 |
| **Gender**（all female） | 26 | 26 | 1 |
| **ECOG PS** |  |  | 1 |
| 0-1 | 18 | 18 |  |
| 2 | 8 | 8 |  |
| **Differentiation** |  |  | 0.905 |
| Luminal A | 8 | 8 |  |
| Luminal B | 8 | 9 |  |
| Her-2 (+++) | 6 | 6 |  |
| Triple negative | 4 | 3 |  |
| **T stage** |  |  | 0.915 |
| 1 | 3 | 3 |  |
| 2 | 6 | 6 |  |
| 3 | 8 | 7 |  |
| 4 | 9 | 10 |  |
| **N Stage** |  |  | 1 |
| 0 | 10 | 9 |  |
| 1 | 7 | 8 |  |
| 2 | 3 | 4 |  |
| 3 | 6 | 5 |  |
| **M Stage** |  |  | 1 |
| 0 | 26 | 26 |  |
| **Clinical Stage** |  |  | 0.822 |
| II | 6 | 5 |  |
| III | 16 | 17 |  |
| IV | 4 | 4 |  |
| **Chemotherapy** |  |  | 1 |
| Yes | 16 | 16 |  |
| No | 10 | 10 |  |
| **Previous RT** |  |  | 1 |
| Yes | 10 | 10 |  |
| No | 16 | 16 |  |

LDR, low-dose radiation; RT, radiation therapy

**Table 2 The acute side effects of two group patients**

|  | **0** | **I** | **II** | **III** | **IV** |
| --- | --- | --- | --- | --- | --- |
| **Dermatitis** |  |  |  |  |  |
| LDR Group（n） | 0 | 18 | 8 | 0 | 0 |
| No LDR Group （n） | 0 | 7 | 16 | 3 | 0 |
| *p* |  | **0.002** | **0.026** | 0.077 |  |
| **Soft tissue injury** |  |  |  |  |  |
| LDR Group（n） | 2 | 21 | 3 | 0 | 0 |
| No LDR Group （n） | 1 | 10 | 14 | 1 | 0 |
| *p* | 0.556 | **0.002** | **0.001** | 0.317 |  |
| **Granulocytopenia** |  |  |  |  |  |
| LDR Group（n） | 5 | 12 | 7 | 2 | 0 |
| No LDR Group （n） | 4 | 7 | 6 | 7 | 2 |
| *p* | 0.717 | 0.150 | 0.510 | 0.070 | 0.153 |
| **Anemia** |  |  |  |  |  |
| LDR Group（n） | 13 | 7 | 5 | 1 | 0 |
| No LDR Group （n） | 12 | 4 | 8 | 2 | 0 |
| *p* | 0.781 | 0.308 | 0.337 | 0.552 |  |
| **Thrombocytopenia** |  |  |  |  |  |
| LDR Group（n） | 14 | 10 | 2 | 0 | 0 |
| No LDR Group （n） | 14 | 5 | 7 | 0 | 0 |
| *p* | 1 | 0.126 | 0.070 |  |  |
| **Swallowing discomfort** |  |  |  |  |  |
| LDR Group（n） | 8 | 18 | 0 | 0 | 0 |
| No LDR Group （n） | 6 | 16 | 4 | 0 | 0 |
| *p* | 0.532 | 0.846 | **0.039** |  |  |
| **Nausea** |  |  |  |  |  |
| LDR Group（n） | 8 | 18 | 0 | 0 | 0 |
| No LDR Group （n） | 12 | 13 | 1 | 0 | 0 |
| *p* | 0.358 | 0.158 | 0.317 |  |  |

LDR, low-dose radiation

**Table 3 Correlation analysis between side effects and LDR in patients who have received Previous radiotherapy**

|  |  | **Previous RT** | |
| --- | --- | --- | --- |
|  |  | **LDR Group** | **No LDR Group** |
| **Dermatitis** | *x*^2^ | 0.885 | 0.849 |
|  | *p* | 0.347 | 0.033 |
| **Soft tissue injury** | *x*^2^ | 0.147 | 8.617 |
|  | *p* | 0.929 | 0.035 |
| **Granulocytopenia** | *x*^2^ | 13.677 | 9.502 |
|  | *p* | 0.003 | 0.050 |
| **Anemia** | *x*^2^ | 3.659 | 1.530 |
|  | *p* | 0.301 | 0.675 |
| **Thrombocytopenia** | *x*^2^ | 0.529 | 0.707 |
|  | *p* | 0.409 | 0.815 |
| **Swallowing discomfort** | *x*^2^ | 6.518 | 4.523 |
|  | *p* | 0.011 | 0.104 |
| **Nausea** | *x*^2^ | 7.222 | 7.583 |
|  | *p* | 0.007 | 0.023 |

LDR, low-dose radiation; RT, radiation therapy

**Table 4 Recent efficacy analysis of two groups patients**

|  | **CR** | **PR** | **SD** | **PD** |
| --- | --- | --- | --- | --- |
| **LDR Group（n）** | 11 | 14 | 1 | 0 |
| **No LDR Group （n）** | 10 | 13 | 3 | 0 |
| ***p*** | 0.777 | 0.781 | 0.303 |  |

LDR, low-dose radiation; CR, complete response; PR, partial response; SD, stable disease; PD, progressive disease
