# Supplementary figures and images for: Effect of low-dose radiation pre-irradiation on postoperative local chest wall recurrence of breast cancer—A retrospective study
Source: PeerJ. 2025 Jan 2;13:e18717. doi: 10.7717/peerj.18717 (PMC11700491; doi:10.7717/peerj.18717)

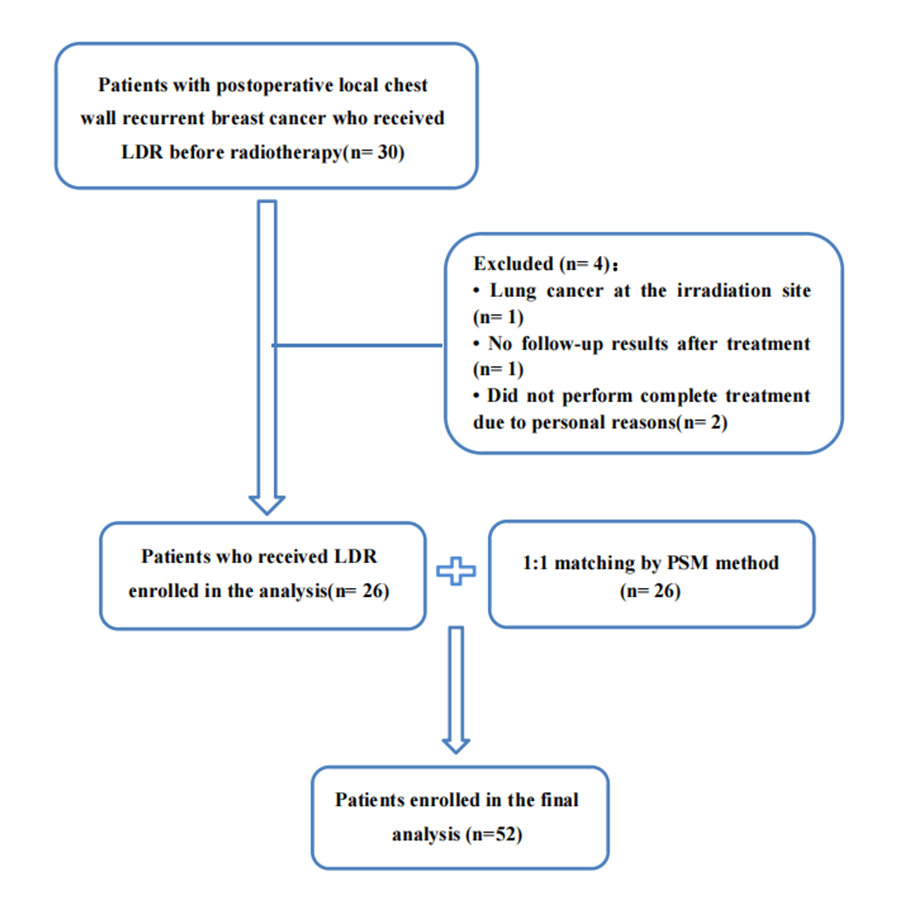

Supplement: Supplemental Information 1 [file peerj-13-18717-s001.zip › data/Figure 1/FIG 1.png]

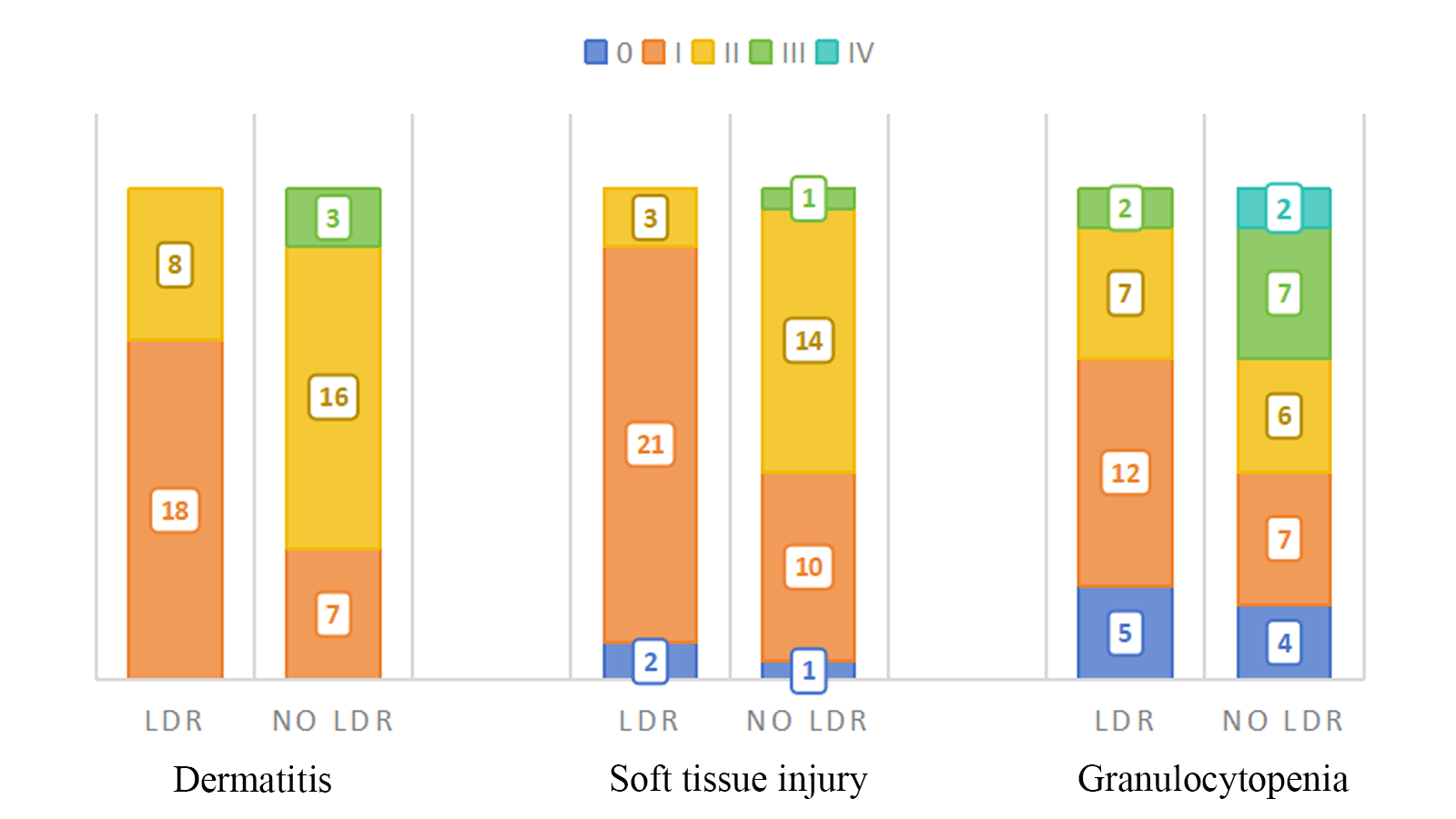

Supplement: Supplemental Information 1 [file peerj-13-18717-s001.zip › data/Figure 2/FIG 2.png]

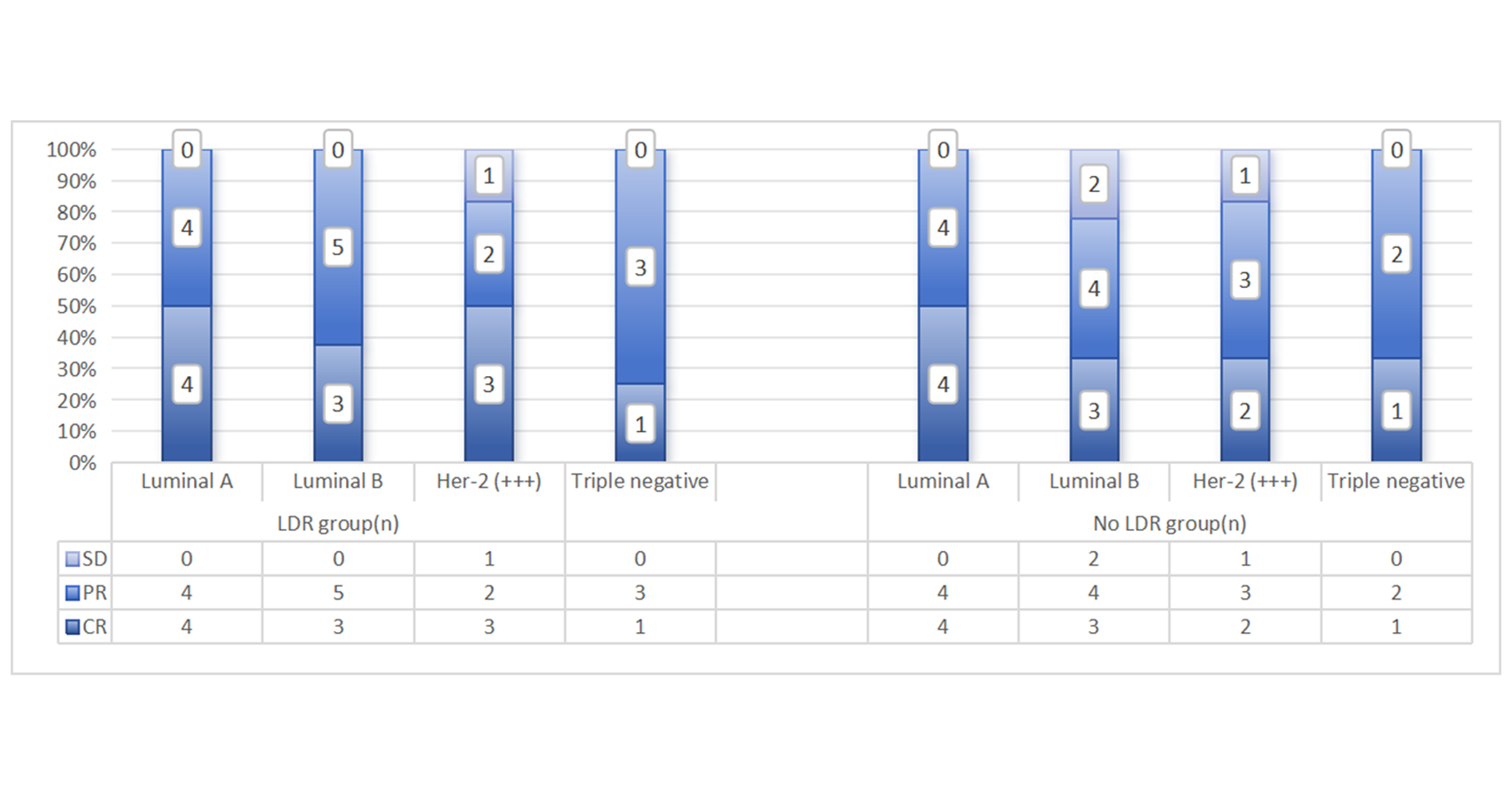

Supplement: Supplemental Information 1 [file peerj-13-18717-s001.zip › data/Figure 3/FIG 3.png]

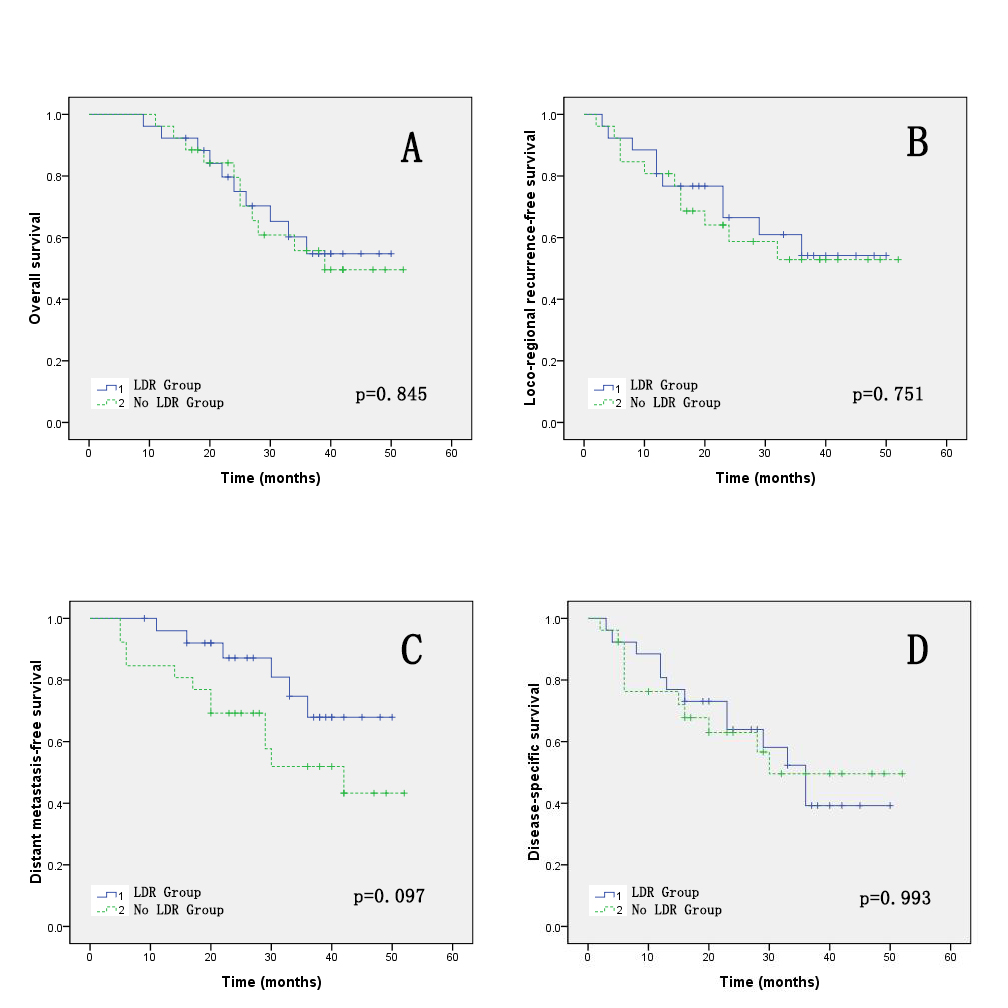

Supplement: Supplemental Information 1 [file peerj-13-18717-s001.zip › data/Figure 4/FIG 4.png]
