# Supplementary material for: Effect of low-dose radiation pre-irradiation on postoperative local chest wall recurrence of breast cancer—A retrospective study
Source: PeerJ. 2025 Jan 2;13:e18717. doi: 10.7717/peerj.18717 (PMC11700491; doi:10.7717/peerj.18717)
Supplement: Supplemental Information 2 [file peerj-13-18717-s002.docx]

**Codebook for categoriacal data**

Group

1= LDR group; 2= No LDR group

ECOG for ECOG Level

1= Lever 1; 2= Lever 2

T, N, M stage and C stage for Clinical stage

1= Stage 1; 2= Stage 2; 3= Stage 3; 4= Stage 4

Differentiation

1 = Luminal A; 2 = Luminal B; 3 = Triple negative; 4 = Her-2 (+++)

C for chemotherapy

0= No; 1= Yes

PRT for primary radiotherapy

0=No; 1= Yes

Skin for dermatitis

0 = grade 0; 1= grade 1; 2= grade 2; 3=grade 3; 4= grade 4

Soft for Soft tissue injury

0 = grade 0; 1= grade 1; 2= grade 2; 3=grade 3; 4= grade 4

Leukocyte for Granulocytopenia

0 = grade 0; 1= grade 1; 2= grade 2; 3=grade 3; 4= grade 4

Erythrocyte for Anemia

0 = grade 0; 1= grade 1; 2= grade 2; 3=grade 3; 4= grade 4

Platelet for Thrombocytopenia

0 = grade 0; 1= grade 1; 2= grade 2; 3=grade 3; 4= grade 4

Esopharyngeal for Swallowing discomfort

0 = grade 0; 1= grade 1; 2= grade 2; 3=grade 3; 4= grade 4

Nausea

0 = grade 0; 1= grade 1; 2= grade 2; 3=grade 3; 4= grade 4

Effect

1= CR (Complete Response); 2= PR (Partial Response)

3= SD (Stable Disease); 4=PD (Progressive Disease)

OS for overall survival

0= Alive; 1=death

OS time:for the date of initial treatment until last follow-up or death

LRFS for loco-regional recurrence-free survival

0= Alive; 1=death

LRFS time: the date of the first chest wall locoregional recurrence, or death from any cause, or last follow-up

DMFS for distant metastasis-free survival

0= Alive; 1=death

DMFS time: the date of initial treatment until the date of first distant metastasis, or death from any cause, or last follow-up

0= Alive; 1=death

DSS for disease-specific survival

DSS time: the date of initial treatment until death due to breast cancer, or last follow-up

the date of initial treatment to (DMFS) was defined as the date of initial treatment until the date of first distant metastasis, or death from any cause, or last follow-up. Disease-specific survival (DSS) was defined as the date of initial treatment until death due to breast cancer, or last follow-up.
